# Performing for Better Communication: Creativity, Cognitive-Emotional Skills and Embodied Language in Primary Schools

**DOI:** 10.3390/jintelligence11070140

**Published:** 2023-07-14

**Authors:** Sandrine Eschenauer, Raphaële Tsao, Thierry Legou, Marion Tellier, Carine André, Isabelle Brugnoli, Anne Tortel, Aurélie Pasquier

**Affiliations:** 1 Aix-Marseille Univ, CNRS, LPL, 13100 Aix-en-Provence, France; 2 Aix-Marseille Univ, Pôle Pilote AMPIRIC, 13013 Marseille, France; 3 Institute of Creativity and Innovation from Aix-Marseille Univ—InCIAM, 13100 Aix-en-Provence, France; 4 SFERE-Provence, 13013 Marseille, France; 5 Aix-Marseille Univ, Institute for Language, Communication and the Brain, ILCB, 13100 Aix-en-Provence, France; 6 Aix-Marseille Univ, PSYCLE, 13100 Aix-en-Provence, France; 7 University Paris-Est Créteil, IMAGER—Languenact, 94100 Créteil, France; 8 Aix-Marseille Univ, ADEF—GCAF, 13013 Marseille, France

**Keywords:** creativity, emotional skills, communication, executive functions, performative theatre/drama, embodiment, English as foreign language (EFL), modern language didactics (MLD), inclusive education (EI), primary school

## Abstract

While the diversity and complexity of the links between creativity and emotional skills as well as their effects on cognitive processes are now established, few approaches to implementing them in schools have been evaluated. Within the framework of the enactive paradigm, which considers the complexity and dynamics of language as a cognitive process, we study how an approach based on performative theatre can synergistically stimulate creativity (artistic, bodily and linguistic), emotional skills (identifying and understanding emotions) and executive functions (especially inhibition, cognitive flexibility and emotional control), all as components defined in the context of oral communication. Stimulating this synergy in the context of foreign language teaching may be especially beneficial for children with communication disorders. This paper presents the first results of the CELAVIE pilot study (Creativity, Empathy and Emotions in Language learning with Autism for an Inclusive Education) through a case study of a pupil with a neurodevelopmental disorder included in a 4th-grade class. The results show a progression in oral communication in English as a Foreign Language (EFL), in emotional skills and creativity.

## 1. Introduction

“Creativity is a cognitive-emotional-manipulative experience that is accessible to all people. Creativity is cognitive because it is about innovating and developing ideas and occurs via specialized mental processes. It is emotional because emotions are integral and ‘loom large’ in the creative process”.

(Gnezda 2011, p. 47)

The diversity and complexity of the links between creativity and emotional skills as well as their effects on cognitive processes are now established (Besançon and Lubart 2015; Gnezda 2011; Lubart et al. 2015). We also know that there are complex links between emotions and language (Beck et al. 2012; Capron Puozzo and Piccardo 2013; Pasquier et al. 2022; Pons et al. 2003). Furthermore, creativity and language are closely related (Aden 2009; Cook 2000; Eschenauer 2019a; Jones 2015). It therefore seems interesting to explore the links between emotions, creativity and language as cognitive processes. However, few studies conducted in schools have assessed these links.

The main aim of the CELAVIE feasibility study (*Creativity, Empathy and Emotions in Language learning for an Inclusive Education*) presented here is to measure the effects of the drama-based performative approach on the oral communication skills of primary school students in English as a Foreign Language (EFL) in pupils with and without neurodevelopmental disorders (NDDS) as an inclusive goal. In this article, we explore through a case study of a student with NDDS how a performance theatre approach can develop communication through creative enactment, cognitive-emotional skills and oral production skills.

Like the majority of Western countries, France has followed the inclusive school model since the Salamanca Declaration (United Nations Educational, Scientific and Cultural Organization [UNESCO] 1994), which defines both what Special Educational Needs are and the means of action to be implemented. Educational policies in France (Ministère de l’éducation nationale et de la jeunesse 2005, 2013) have thus enabled children with disabilities to have easier access to schooling in a mainstream environment. Indeed, the latest report by the French Ministry of Education and Research’s Department of Evaluation, Forecasting and Performance [DEPP] (Dauphin 2022) indicates that since the 2005 law, the number of children with disabilities enrolled in mainstream schools has risen by 6–7% per year. At the start of the 2021 school year, this involved almost 476,000 children or adolescents. On this subject, the report by the French National Council for School Evaluation (Conseil national d’évaluation du système scolaire [CNESCO] 2016) sets out a series of recommendations likely to promote the inclusion of pupils with disabilities. The role of collaborative practices and the issues surrounding training are put forward as key levers for inclusive schools.

We choose here to focus on a case study of a child with NDDS, that will allow us to explore the dynamics of cognitive-emotional and creative processes and how they relate to one another. For Lubart et al. (2015), creativity is defined as “the ability to produce something that is both novel and appropriate to the context in which it occurs” (p. 23). Furthermore, cognitive-emotional skills include the concept of emotional intelligence (Mayer and Salovey 1997), taken here in terms of emotional competencies rather than personality traits (Sander and Scherer 2019). There are complex links between creative and emotional processes (Lubart et al. 2015), which have been studied in different fields related to learning. We have chosen to study them in relation to communicative processes, in line with work carried out within the enaction paradigm (Varela 1989), applied in the context of foreign language learning (Aden 2009; Eschenauer 2019a, 2019b). Creativity and emotional skills are thus addressed in our study in a holistic, dynamic and integrative approach to modern foreign language learning processes in the context of inclusive education (EI). Our approach differs from more traditional acquisitional approaches that consider the components of communication (lexicon, syntax, prosody, interactions, body language, emotions, etc.) separately.

Varela proposes an integrative model of communication that is based on the complexity and dynamics of *languaging* as an embodied cognitive and creative process. “Embodied” includes, for example, emotions, posture, sensoriality, gestures, etc. For researchers in this field, there is no gap between the body and the mind (Noe 2016; Varela 2017). Thus, communication is not only information sent from a sender to a receiver, nor purely linguistic content consisting of lexicon and syntax. From an enactive perspective, language creativity is hence mainly concerned with the ability of speakers to adapt their behaviour and to produce new language appropriate to the context, but also with the originality of students’ communicative proposals (Aden 2009). Communication is a creative act since it allows for the infinite combination of a finite number of words and reinvents itself over time. Cook (2000) combines language manipulation with language play and language creativity. For him, EFL learners are using language creatively when they play with language forms, with semantic and pragmatic elements. For Carter and McCarthy (2004), pattern reformulation is also creative. Indeed, speakers manipulate existing patterns of prefabricated language to construct new creative sequences. Authors interested in the importance of creativity in foreign language acquisition agree on including humour, banter and joking as creative acts of language manipulation (Cook 2000; Kramsch 2009; Bell 2012). Hence communication is in itself a creative process, both in its language nature and from a behavioural point of view. Results from national and international studies show that creativity could be a lever for better EFL learning (Eschenauer 2019a; Jones 2015; Richards 2013)—and as many studies have shown, creativity can be learned (Bonnardel et al. 2023; Capron Puozzo 2016), for example, by practising acting (Hainselin et al. 2018).

Drama practice as an emotional and creative approach to language teaching can develop communication skills in EFL. It seems to be one of the best methods, as it balances thoughts and feelings (Gabitova et al. 2018). Yet, this approach is at odds with standard teaching practices in elementary schools. Indeed, primary school teachers focus more on the acquisition of lexicon and syntax and they most often decontextualise lessons (Dawoud et al. 2020). These practices have limitations that have been widely pointed out for the last thirty years. New approaches in France, such as role play (Care and Debyser 1991), were first proposed empirically and then researched and transformed into drama (Aden and Lovelace 2004), as remembered by Eschenauer et al. (2023). The positive effects of drama practice on foreign language learning have been noted (Abdelkader et al. 2013; Sambanis 2013; Schewe 1993), especially in relation to creative processes and emotional skills (Aden and Lovelace 2004). Effects of EFL instruction through drama have also been shown on prosodic production (Thienkalaya and Chusanachoti 2020). Furthermore, recent studies based on communities of practice highlight the contribution of drama-based practice to the development of social skills (Andréoléty 2015; Amonkar et al. 2021; Cook 2020; Corbett et al. 2019; Drill and Bellini 2022; Ioannou et al. 2020), basic learning (Mpella et al. 2019; Saint Martin (de) and Astier 2021) and communication skills in children with NDDS (Kumar and Raja 2009).

**Figure 1:**
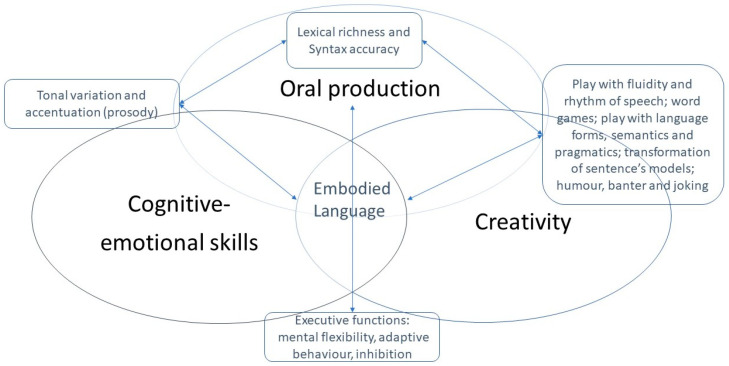
Integrative model from the CELAVIE study of three major components of oral communication.

In view of these considerations, it now seems important to refer to definitions of communication in Modern Language Didactics (MLD) that integrate its complex and dynamic nature, including creativity and cognitive-emotional skills. This is why we develop in this paper an integrative model presenting what we believe to be three major components of oral communication (Figure 1). We assume that creativity and emotional literacy are both means (in the sense of processes) and communicative goals to be developed, as well as oral production.

In this model, we can see that oral production depends on cognitive-emotional skills and creativity and that embodied language lies at the intersection of these three elements.

We have already pointed out that there is ample evidence of the complex links between emotions and creativity (Lubart et al. 2015). From a functionalist point of view, experiencing different emotional states (both positive and negative) is thought to enhance creativity (Abele-Brehm 1992; Zenasni and Lubart 2008). Executive functions are central to the psychological development of children, as they must learn to constantly adjust their behaviour to internal and environmental contingencies. This constant adjustment involves emotional regulation (Roy 2015). Mental flexibility underlies these behavioural and sensitive adaptations. The emotional state experienced could generate a level of arousal experienced as uncomfortable, which can be reduced by investing in creative activities (Adaman and Blaney 1995). Executive functions allow for the functioning of cognitive-emotional skills such as creativity. They are also needed to produce language (Roy 2015).

As emotional intensity, emotional expressiveness and emotional awareness (which refers to the abilities to perceive and understand emotions; Lane and Schwartz 1987) are positively correlated (Pasquier et al. 2009), it seems important to value didactic approaches that elicit strong emotions, their expression, and their (conscious) perception in connection with EFL—as possible, for example, through theatre. A motivating and innovative task such as a drama-based performance in a school context could evolve emotional awareness and creative engagement for the development of communicative skills in EFL.

Here we present a case that will be studied according to our three-component model of communication (see Figure 1). We chose the case of a child with NDDS who presents specifically communication disorders. We will explore how a performance theatre approach can develop communication through creativity, cognitive-emotional skills and oral production skills.

## 2. Materials and Methods

### 2.1. Design and General Procedures

#### 2.1.1. A Test-Retest Research Design

We measured the evolution of three components of communication (cf. Figure 1) before and after the implementation of a performative theatre approach. The methodological approach of our research is interdisciplinary because we have developed a psycho-phenomenological methodology in accordance with the enaction paradigm (Varela 1996), which frames CELAVIE.

The study presented in this paper is based on the data collection of the feasibility phase of the larger CELAVIE project. This feasibility phase took place over 6 months, including one and a half months for the inclusion and pre-testing phases, 4 months of experimentation and 15 days for post-testing. The experimental approach is the practice of performance art (drama) in English for EFL learners.

Our data obtained through qualitative and quantitative measurement tools (self-reported and hetero-reported data, audio-video recordings) were qualitatively processed in a single case study.

We focus here on data related to cognitive-emotional skills (in particular emotional awareness and mental flexibility), which we link to the analysis of body language (especially body engagement), foreign language use and the creativity indicators of Fortin et al. (2014). The qualitative analysis from the interviews is based on content analysis (Bardin [1977] 2013; Mayring 2010). Coding for content analysis was performed double-blind with inter-rater agreement.

#### 2.1.2. The Drama-Based Performative Approach

The students had been learning English at school for three years, for 1.5 h a week (pre-A1 level, starters). For our experimentation, an English-speaking actor set up 4 performative theatre workshops divided into 8 sessions of 2 h, i.e., 16 h over a period of 4 months. Both the actor and the teacher worked with the whole class, but activities could be performed in full class groups, half-groups, small groups of 3 or 4 or by a student alone. The teacher articulated these workshops with classroom language activities twice a week, in sessions of 30 min to 1 h, also based on the characteristics of performance (movement, space, voice, speech, etc. Examples of activities in the classroom were: presentation games with skits, vocabulary of emotions by acting out the emotions, then adding a sentence, etc.). In this way, language elements such as lexicon and syntax that had emerged during the creative drama activities could be reworked and formalised in the classroom. The EFL embodied experience was complemented by an awareness of how this new language works. The medium that linked the workshops to the classroom activities and from which the actor drew stories of encounters between the students was the children’s book *Voices in the Park*, by Anthony Browne. We chose this book for its aesthetic and creative proposition, as well as for the content related to our research questions. Indeed, it is a story about a walk in a park told by four different characters with four different emotions. Each story generates a different vision of the world, and the illustrations transform the park according to the subjective emotional experience of the narrator. This book is, therefore, very phenomenological. It allows us to work on the awareness of the characters’ emotions and the acting with them that links the children’s embodied knowledge (self-centred point of view) to reflection from the perspective of others (hetero-centred point of view). The didactisation of this book, originally written for native speakers, was not achieved by simplifying the text as teachers usually do, but through theatrical practice based on the illustrations and the decoding of the characters’ emotions.

Performative theatre differs from more canonical theatre practice in its emergent and relational nature. The theatre we call “canonical” is a stage interpretation of a text. The production is more linear: writing (often by a third person), learning and interpreting. Performative theatre is closer to stage creation, except that it can be performed in any space: in the street, in a museum, in a café, etc. Besides, it has always had an unpredictable dimension since the story is transforming itself through the relationship with the audience and the space. Performative theatre could be described with fractals (i.e., not linear): writing comes after the exploration of the space and the sharing of creative experiences and stories between participants. In a school setting, the audience may be part of the class, students from other classes, families, or even a wider audience for larger projects.

Performance is an embodied and phenomenological “social act” (Schechner 2003). Hence, performance also has an aesthetic dimension that engages the sensory and emotional experience of everyday life—but exacerbates it in its concentrated artistic form. This is why we choose this practice in schools: it brings everyday “real life” and creativity into the classroom rather than reproducing didactic schemes that disembody language and ignore its creative nature. The performative approach is not limited to theatre workshops. It articulates moments of workshop with moments of formalisation to work on the text, on the foreign language, and moments of reflexivity. The students explain what they have experienced and understood both from their own history and from what happened in the class or in the workshop between them. This reflexive aesthetic co-construction makes it possible to introduce informal forms of learning into the formal school setting (Eschenauer 2019b).

**Figure 2:**
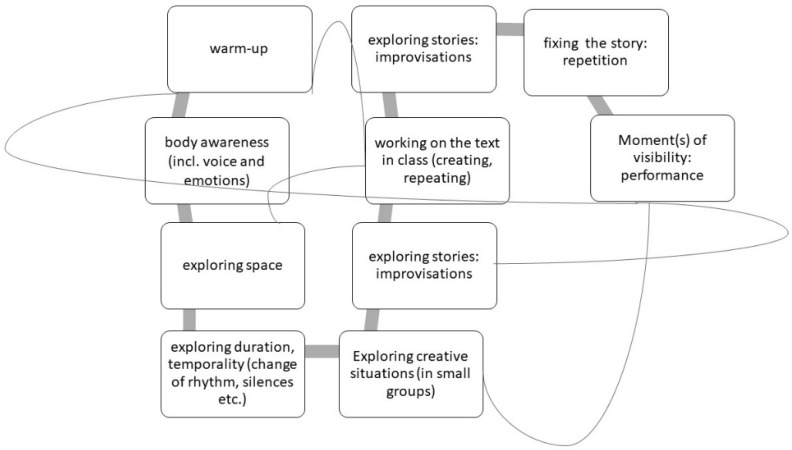
Proposal of a dynamic relationship model of the basic elements of a performative approach to foreign language didactics.

There is consequently no clear linear pattern for a performative approach in EFL teaching but rather a dynamic approach with some recurrent ingredients (warm-up, exploring spaces, working on the text, etc.), as we propose to schematise in Figure 2.

### 2.2. Participant: A Case Study

We choose here to present the case of Charles, aged 10 years and 6 months, who attends a mainstream school. He is attached to a reference class of his age group and benefits, according to his needs, from shared support time within a group of children with special needs (local unit for school inclusion, ULIS). He has been followed up in a medical-psychological-pedagogical centre for several years in speech therapy, psychomotricity and psychology. Charles has a neurodevelopmental disorder (NDD) characterised by a limitation in cognitive functioning and adaptation capacities. He has no language problems apart from a certain slowness of production. He is able to understand a variety of texts even though his fluency level is well below his age group. He can produce complex sentences and listen attentively to messages or instructions from an adult or a peer but has more difficulty in playing with the volume of his voice and posture, in role-playing and debating. He could benefit from shared English time in the ordinary class. In terms of cognitive skills, Charles has difficulty in learning situations that require planning and mental flexibility. His engagement in the activity is characterised by a certain slowness. On the other hand, he performs relatively well in tasks requiring sustained attention and memorisation. In view of his disorder, he has good adaptive behavioural skills. He needs a support worker to provide material assistance in situations that present a risk of cognitive overload, such as those requiring planning or mental flexibility.

### 2.3. Materials and Data Processing Procedure

Using a test-retest design, we measure the effects of a performative teaching approach on Charles’ communication skills, bearing in mind that oral communication relies on cognitive-emotional skills and creativity to develop oral production (through embodied language). Table 1 summarises the variables (and data collection tools) collected at the various stages of the study.

#### 2.3.1. Executive Functions Assessment Procedure

We assessed the executive functions by scoring the teacher version of the Behaviour Rating Inventory of Executive Function (BRIEF, teacher version; Gioia et al. 2013). This inventory is an evaluation tool for ecological analysis of the executive functioning of children and adolescents through its repercussions in everyday school life.

In our study, we had to abandon the parents’ version, which posed the double difficulty of language (some parents have no or little command of the French language) and complexity (some parents themselves have cognitive disorders that prevent them from answering the questions). This questionnaire makes it possible to identify, in an ecological way, the executive functioning of children and adolescents.

This inventory is composed of 86 questions grouped into 8 scales, allowing the calculation of two indices: the behavioural regulation index and the metacognition index. The reliability indices indicate good internal consistency, with Cronbach’s alpha coefficients ranging from 0.80 to 0.97. Regarding construct validity, executive functions, assessed by the BRIEF, correlate with a set of other behavioural measures (Gioia et al. 2013).

Regarding the objective of the project, we focused our analyses on the three scales (inhibition, flexibility and emotional control) that allow us to calculate a behavioural regulation index score. To be precise, the scores obtained represent degradation scores, i.e., the lower the scores, the better the performances evaluated.

#### 2.3.2. Cognitive-Emotional Skills Assessment Procedure

Cognitive-emotional skills are assessed through the Levels of Emotional Awareness Scale for Children, LEAS-C (Bajgar et al. 2005; Lane and Smith 2021), translated into French by the authors of the test themselves. This version has already shown its discriminatory qualities (Pasquier et al. 2022). It is a tool designed to assess the emotional awareness of children aged 8 and over. It consists of 12 scenarios, based on everyday social situations, each one involving two people, oneself and another person. Each participant is invited to reply, indicating what they and the other person could feel in each situation. (How would you feel? How would the other person feel?). The scenarios are based on four emotions (from the basic emotions described by Izard (1977): anger, fear, happiness and sadness. Each one is presented in three different situations in a mixed order. We decided to use an adapted version of the scale with only six scenarios selected from the 12 initially provided (Veirman et al. 2016). On the one hand, this allows the testing time to be reduced, and on the other hand, the adapted version also proves to be more reliable as only the most sensitive items are retained (Veirman et al. 2016). The scale’s rating is based on the complexity of the vocabulary related to emotions and their differentiation from each other by respondents. This questionnaire does not have a normative aim insofar as there are no expected “correct” responses, both from the point of view of form (grammatical and/or spelling errors) and substance. It is used to calculate three scores for each participant: an overall emotional awareness score (LEAS-T) and two subscores for intra-subjective emotional awareness (LEAS-S) and intersubjective emotional awareness (LEAS-O). The writing time does not exceed 20 min. If students have writing difficulties, they can tell an adult what they want to write.

We believe it is important to combine auto- and hetero-declarative assessment tools (LEAS-C, BRIEF) at pre- and post-test with behavioural observations (via video recordings). This allowed us to assess creativity through executive functions, cognitive-emotional scores, language embodiment and oral production.

#### 2.3.3. Embodied Language: Body Involvement and Behaviour Coding Procedure

In order to better observe the links between executive functions, cognitive-emotional skills, creativity, and embodied language, we decided to code the participants’ language behaviour using the indicator of bodily engagement in the activity. We chose to code mainly sessions 1 (discovery, icebreaker) and 2 (first creative situations based on the children’s book *Voices in the Park*) and the final sessions (sessions 7: last improvisation and fixing of the stories invented by each group of pupils and 8: moment of visibility) of the theatre workshops to observe the evolution of the student’s body involvement.

Body involvement in the performative theatre activity was coded on Elan software (Sloetjes and Wittenburg 2008). A coding scheme of 4 labels was developed for this specific analysis: (1) “out of space”: when the participant leaves the space of the activity; (2) “in space but motionless”: when he is in the space of the activity but does not participate; (3) “in space with unexpected behaviour”: when he is in the space but acts differently from what is expected; and (4) “in space with expected behaviour”: when he is in the space and acts as expected.

Sessions 1, 2 and 7 were entirely coded by one coder, then Sessions 1 and 7 were counter-coded by another coder following Tellier’s method (Tellier 2014). Both codings were then compared (agreement reached 65%). The 35% of disagreement can be explained by the fact that the definition of label 3 (“in space with unexpected behaviour”) had to be clarified since it was sometimes misinterpreted by one of the coders. Every disagreement was discussed by both coders to determine which final label to adopt and reach total agreement. Our coding manual was also corrected for future data coding.

Finally, behavioural coding was also performed by triple-blind viewing of the videos. We focused our observation of Charles’ behaviour on two points: his behaviour in relation to himself and in relation to others. For his relationship with himself, we focused on the development of his spontaneity in creative activities (in the workshops with the actor). For the quality of individual behaviour, we defined three gradations: passive (accepts peer suggestions), semi-autonomous (indicates to a reference adult that he would like to make suggestions), and autonomous (makes spontaneous suggestions to peers, bodily and/or verbally). In terms of his relationship with others, we noted his tendency to collaborate and to construct the scenes by adapting his behaviour to the group’s proposals. Our observations were complemented by the teacher’s report on the evolution of Charles’ behaviour in class during the learning of English, which we coded using thematic discourse analysis. This coding allowed us to compare the evolution of the participant’s bodily involvement with the evolution of his oral production and creative proposals.

#### 2.3.4. Oral Production Coding Procedure

To analyse the evolution of Charles’ language production, we conducted a qualitative study of the prosodic curve variation. We analysed the development of Charles’ oral competence by comparing his production in pre- and post-test foreign language. The evaluation criteria are based on the evolution of the prosodic production. We also quantified Charles’ EFL oral production in the pre- and post-test and analysed the degree of autonomy of his production. Prosody informs us elsewhere of the degree of awareness of emotional situations experienced or interpreted by the student. Prosody is an essential component of oral language because it plays an important role in the vocal communication of emotions that give meaning to speech (Petrone et al. 2016). Indeed, although the channels are different, the emotional perception of speech is directly related to the semantic processing of speech (Ben et al. 2016; Shakuf et al. 2022). Prosody facilitates the decoding of the semantic content of spoken language, as it can be used both for the recognition of the emotional content of speech (e.g., by increasing the speech rate or intonation of speech) and for linguistic processing (e.g., to disambiguate syntactic information).

Finally, we relied on the teacher’s testimony given at the end of the experimentation of the feasibility phase describing his academic evolution in English production, as well as the observation of audio-video recordings made during the drama sessions 1 (discovery, icebreaker), 2 (first creative situations based on the children’s book *Voices in the Park*) and 7 (last improvisation fixing the stories invented by each group of pupils). The EDA data can be used to complement the analysis performed by blind counter-coding. If the coding from the observation of the videos shows a change in attitude (body involvement) and a change in the use of English (see Figure 1 for the indicators: lexical richness and syntax accuracy; play with fluidity and rhythm of speech; word games; play with language forms, semantics and pragmatics; transformation of sentence’s models; humour, banter and joking) and/or in the prosody (tonal variation and accentuation), it is possible to go and check the electrodermal activity and observe whether indicators of strong emotions such as stress, joy, etc. appear too (peaks and valleys). An interview with the pupil about this moment in time provides the perceptual data necessary for the final interpretation.

#### 2.3.5. Creativity

To study the development of the student’s creative skills, which is the subject of our case study, we first measure their evolution through the discourse analysis of the teacher’s and the actor’s testimonies after the experimentation, focusing on the linguistic aspects (body, verbal language) and on Charles’ scenic proposals (observed through the video recordings). The actor’s testimonial was written after having seen the videos from sessions 1, 2 and 7, 8. So she could remember what she observed directly during the sessions and indirectly analyse the videos.

In a second step, we triangulate all the data presented for this article and synthesise the results in a table. The indicators of Charles’ creative skills are taken from Fortin et al.’s (2014) work on assessing creative skills in dance as a performance art. Thus, this framework provides valuable indicators for analysing the development of Charles’ creative skills in theatre performance. Fortin and her colleagues propose 5 components to measure the development of the creative process in the performance arts. These 5 components are broken down into indicators, which we have used as an analysis tool to position all the data from the different collection tools on the markers of creativity. We complete with a 6th component relating to a verbal dimension specific to drama-based performance as we practice it in the experiment:

- Quality of presence to oneself, to the world, to experience (physical, emotional and intellectual availability; imagination; listening, observation; acceptance of the new, of the unknown).
- Quality of the exploration of postural-mimicry and gestural expression: performance (risk-taking by creating new worlds: ability of students to create and express themselves with the body; appropriation of instructions; commitment).
- Quality of the organisation of time, space and materials for creation (anticipation of the whole project; taking initiatives).
- Quality of collaboration with partners (ability of students to adapt their behaviour; contribution to teamwork; establishing a relationship climate; listening to the ideas of others).
- Quality of reflection on one’s work and the group’s work (analysing one’s own work and that of others; verbalising opinions; consideration of comments and criticism; renewal of one’s representations and perceptions).
- Quality of the formatting of the performance (quality of the verbal and bodily interpretation; selection of material: gestural, verbal, musical, etc.; consideration of technical limitations; harmonisation of the different components of the work).

We added:

- Quality of verbal expression (risk-taking: ability of speakers to produce new language appropriate to the context, originality of the verbal language, i.e., new words or new combinations of words).

Fortin’s and colleagues’ definition of “competence” or creative “skills” meets ours insofar as it means “the mobilisation of resources to resolve situations in an optimal way”. For them, developing one’s creativity and competence in the field of performance arts means,

“on the one hand, developing personal resources that will specifically favour artistic creation and, on the other hand, developing one’s ability to mobilise these same resources in a complex way by engaging in a creative process and in the shaping of artistic and discursive productions” (p. 13).

### 2.4. Links between Cognitive-Emotional Skills and Creativity

The links between cognitive-emotional skills and creativity will be analysed by triangulating the data after detailing each of the measures. In our study, language is at the interface of emotions and creativity. We will therefore end the presentation of our results with a summary of the various indicators relating to cognitive-emotional skills and/or creativity. Taken together, these elements will enable us to draw conclusions about the development of oral communication skills (see Figure 1) in our case study.

## 3. Results

The results show an increase in Charles’s communication skills.

### 3.1. Executive Functions: BRIEF (Teacher Version)

Looking at the pre- and post-test data (Table 2), we can see that Charles’ scores have improved on all the indices. Concerning the behavioural regulation index, which includes three scales: inhibition, flexibility and emotional control, Charles shows more ability to adjust his behaviour in a school context. The change in raw scores on the indices between the pre-test and the post-test is greater than one standard deviation from the French data for boys aged 10 and 11. As Charles is 10 years and 6 months old, we have included both types of data (Table 3 and Table 4).

As we can see in Table 2 on the Inhibition scale, there is a difference of 6 points between the pre- and post-test for Charles, which is more than one standard deviation for the 10 (Table 3) and 11 (Table 4) year-old boys. For the Flexibility Index, the difference between the two test conditions is 3 points, which is also one standard deviation. For the Emotional Control Index, the data show a difference of 7 points, well over one standard deviation. For the Behavioural Regulation Index, the difference between the two conditions is 16 points, or 2 standard deviations. The data, therefore, indicate that Charles made significant progress between the pre-test and the post-test.

Indeed, in view of the observations reported by his teacher, Charles seems to be more at ease in controlling his impulses and behaviours at the right moments in the face of constraints imposed by the teacher in a learning context (inhibition scale).

On the other hand, he was more frequently able to move freely from one situation to another according to the demands of the situation, without necessarily needing support from an adult, showing more flexible behaviour in the post-test situation (flexibility scale). He is also able to modulate his emotions more appropriately (emotional control scale).

These observations converge with the qualitative analyses and his results in the language class, which are improving both in terms of production and in terms of his behaviour when faced with the unknown. We measure these two elements through language tests on the one hand, and the study of the pupil’s bodily engagement on the other, as an indicator of the child’s ability to inhibit negative emotions (such as anger) that usually prevent him from interacting and carrying out activities.

### 3.2. Cognitive-Emotional Skills: LEAS-C

**Figure 3:**
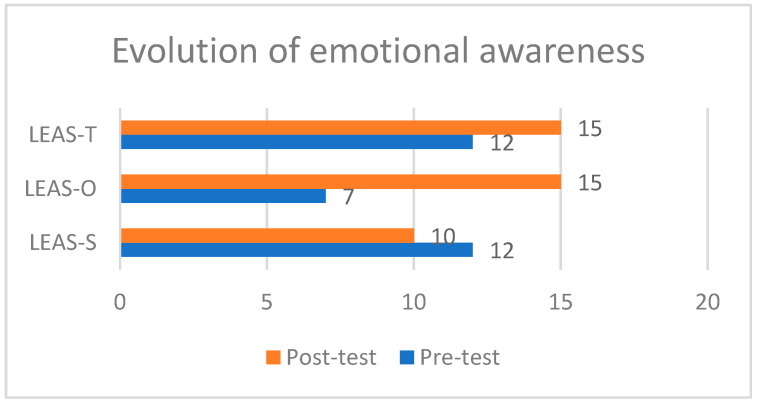
Charles’ LEAS-C scores before et after the performative theatre approach.

Charles’ pre- and post-test LEAS-C scores (Figure 3) show an improvement in inter-individual levels of emotional awareness after training.

In more detail, Charles’ pre-test LEAS-C scores show heterogeneity between intra- and inter-individual levels of emotional awareness. His intra-individual awareness level is average (12 points out of 24), while his inter-individual awareness is well below average (7 points out of 24). He obtained a total score of 12 points out of 30, allowing us to place him at a level of development where the sensory aspects of subjective experience still predominate and the beginning of the translation of the sensible into simple emotional language. It seems that his identification and language expression skills concern mainly personal emotions. The emotions of others are poorly identified and differentiated from internal experience. This clinical picture seems to be consistent with that usually encountered in children with NDDS. Indeed, he has difficulties processing social information, which would explain the difficulties usually found in the expression and recognition of emotional expressions in children with NDDS (see Baurain and Nader-Grosbois 2011 for more details on this subject).

Charles’ LEAS-C scores at the post-test still show heterogeneity, but this time in reverse. The level of intra-individual emotional awareness fell slightly below the average (10 points out of 24), indicating a slight drop in performance during this second phase of the test. However, we are talking here about performance and not ability, insofar as the test conditions were particular (the adult who usually helps him was not present) compared to the pre-test situation. This context could explain his difficulty in focusing on his feelings (which also impacted the total score level, which reached 15 points out of 30 on the post-test). However, his level of inter-individual emotional awareness exceeded the average (15 points out of 24) by doubling the score obtained in phase 1. This time Charles answers all the questions and uses a more precise vocabulary, even if it is a lexicon describing simple emotions. He also seems to be able to consider different emotions in the two protagonists of the same scenario. These results suggest that there was a clear progression in the ability to identify and express the emotions of others between the pre- and post-test LEAS-C. Again, our data are in line with the literature, which points to the need to work on emotional skills in a lively and interactive way in order to make the best progress for the child with an NDD (e.g., Baurain and Nader-Grosbois 2009).

### 3.3. Embodied Language: Body Involvement by Languaging

**Figure 4:**
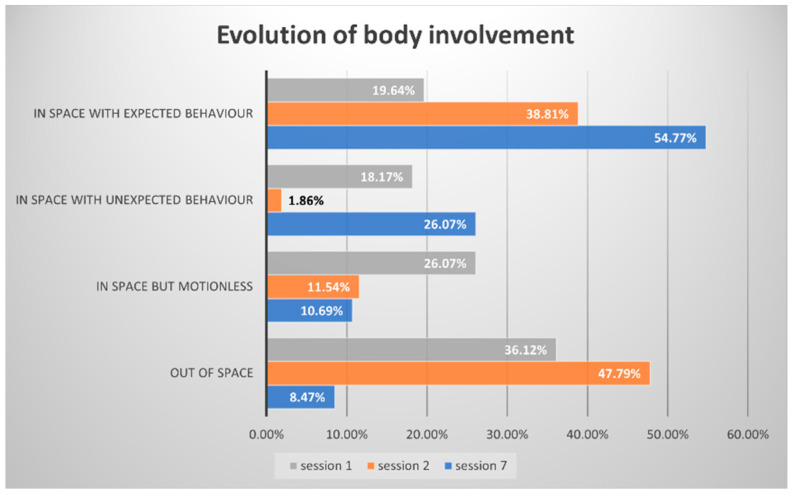
Evolution of body involvement in the performative activity in EFL.

The participant’s involvement in the performance workshop evolves over time. The following chart (Figure 4) presents the duration of each behaviour (out of space, in space but motionless, in space with unexpected behaviour, in space with expected behaviour). In Session 1, the participant’s behaviour was coded for a duration of 2031 s; in Session 2, for 2331 s; and in Session 7, for a total duration of 2776 s. We thus present the descriptive statistics in percentage for a comparison between the 3 sessions.

**Figure 5:**
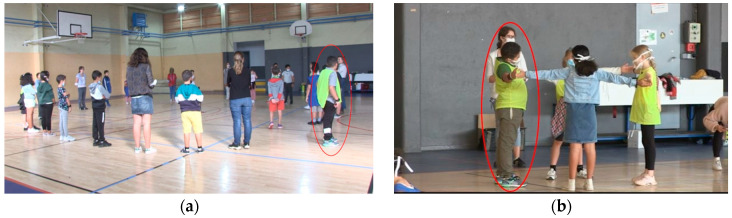
The evolution of body engagement as a result of Charles’ adaptability and as a basis for creative proposals: (**a**) out of space (participant leaves the space of the activity); (**b**) expected behaviour (participant is in the space of the activity and makes creative proposals for staging).

We can see that, in Session 1, Charles spends more than 1/3 of the time out of the space of the activity. It can be moments when he stands out of the circle or even moments when he leaves the centre of the room completely to sit against a wall (see Figure 5a for an example). In Session 2, a large amount of the session is still spent out of the space of the activity (47.79%). This kind of behaviour is considerably reduced over time (only 8.47% in Session 7). His involvement in the performance activities changes too, as the chart shows. The most striking result concerns when he acts as expected (i.e., following the instructions and waiting for his turn): only 19.64% of the time in Session 1, then 38.81% in Session 2 and 54.77% in Session 7 (see Figure 5b for an example).

This analysis could be confirmed through the content analysis of the teacher’s testimonial, which explains the social and cognitive-emotional changes observed in the class after the experiment:

- He is willing to work with other students (he develops social skills).
- He even makes scenic proposals (he develops creativity), both in terms of postural and gestural play, in terms of language creativity.
- And in terms of the emotional interpretation of the character being played (he develops emotional skills). He tells his classmate that he wants to perform his playlet with the anger he feels after a disappointment. He puts this emotion at a distance thanks to the theatrical expression and remains in the language activity.

### 3.4. Creativity

Charles’ creativity increased at the end of the experiment, at the same time as he became more involved in the activities. The cross-analysis of all the measures attempts to highlight the link between the different skills developed: cognitive-emotional, verbal, embodied and creative.

Table 5 represents a synthesis of all the analyses. To complete it, as mentioned in our methodology section, we triangulated the results of the tests with a thematic analysis of the discourse from the interviews with the teacher and the testimony of the actor. The “themes” of the analysis correspond to the 6 components of creativity skills (5 components described by Fortin et al. and the verbal component that we added). In this way, we can analyse and organise our data relating to cognitive (BRIEF analysis) and emotional (LEAS-C) skills, as well as linguistic skills (prosody in the pre- and post-language test + analysis of classroom results on language skills tests, described by the teacher in her testimony) and embodied language (study of Charles’ physical engagement using Elan software). The analysis through the creativity marker shows how drama potentially enabled Charles to progress in cognitive-emotional skills, oral production skills in English and creativity.

### 3.5. Emotions and Creativity

The following Table 6 (Table summarises the main results concerning the development of Charles’ cognitive-emotional skills (BRIEF and LEAS-C) in relation to the development of his creativity skills (Fortin et al.’s framework). The three most important indicators of the 6 creativity indicators analysed previously (see Section 3.4) are shown here. Finally, this table aims to show the role of cognitive-emotional skills and creativity in Charles’ ability to express himself in a modern foreign language.

## 4. Discussion and Conclusions

Our main aim was to explore how a performative theatre tool could develop communication skills in a student with NDDS through creativity and cognitive-emotional skills. Analysis of Charles’ pre- and post-test scores shows a clear progression in cognitive-emotional skills (executive functions and emotional awareness of self and especially others), supported by greater involvement in the performance workshop. This involvement, particularly bodily involvement, in the language produced by Charles is visible through the analysis of behavioural indicators during the performance workshop. Charles’ oral EFL production in class also increased following the implementation of the performative approach. And the intersection of all these data allowed us to make a qualitative analysis of Charles’ creativity. According to the case study here, creativity is a skill developed through performative theatre, and at the same time, it participates in the development of other skills (cognitive-emotional, including verbal skills). This finding is consistent with the work of other researchers who have shown the benefits of drama-based approaches in education for developing various skills related to creativity (Celume et al. 2019).

Once again, we are led to believe that an oral language teaching tool in a natural context of exchange around emotions is effective in developing emotional and language skills in a school context (see Eschenauer 2014, 2019b; Pasquier et al. 2022).

Regarding the use of theatre, the data presented here seem to be in line with previous studies that have shown the effectiveness of drama methods in the development of a second language (Galante and Thomson 2017), communication skills (Gabitova et al. 2018), and the positive effects of a theatre performance on emotional literacy and its link to the development of students’ creativity and EFL skills (Aden and Eschenauer 2020; Eschenauer 2014; Eschenauer and Voise 2017).

More specifically, the case study seems to show that a performative theatrical approach is an interesting modality for the development of socio-emotional and linguistic skills through the initiated and also developed creativity process. Indeed, in the case of Charles, we are led to believe that this performative theatre approach in a school context allowed him to experience a variety of emotions in socially interactive situations for learning EFL. These situations contributed to enriching his understanding of emotions (emotional competence) while promoting a high level of arousal and better regulation of his emotions (greater commitment to activities despite strong emotions such as anger. It could mean that the training may have led to better regulation of emotional processes), leading him to engage in creative activities (Adaman and Blaney 1995). Indeed, the approach does not seem to have had the effect of crossing the threshold of tolerance for too high a level of arousal, which would have made the experience more vulnerable than creative. The framework of the school approach seems to have provided sufficient stabilising and containing aspects to channel the creativity vulnerability factors (Lubart et al. 2015) and allow Charles’ creative communicative production to develop.

Furthermore, Charles’ engagement in creative activities seems to have helped him to internalise the foreign language with which, after the experiment, he allowed himself to play and even generate humorous situations. Before the experiment, Charles reproduced linguistic patterns, but the teachers had never witnessed his independent use of language, let alone the production of humour in English.

This engagement was supported by executive processes such as emotional flexibility, which, as Lubart et al. (2015, p. 37) point out, “is implicated in creativity because it reflects the mobility and flexibility of thinking and the ability and willingness to change registers”. These registers can be linguistic, bodily, emotional or cognitive. In this sense, emotional flexibility (as a “hot” basic executive process) operates in contexts with an affective, emotional and/or motivational component, as defined by Zelazo et al. (2005). Emotional flexibility and creativity could be the engines of communication, especially in foreign languages where it is necessary to change linguistic and cultural referents.

“Everyone has the potential to be creative, but not everyone fulfils that potential” (Runco 2014, p. 39). Charles seems to have been able to do this.

However, at this stage of the study, it is not possible to conclude that the theatrical approach alone could be the source of all of Charles’ progress. Indeed, every child in inclusion progresses because of his inclusion, as shown by various research studies (Gray et al. 2021), and every so-called “ordinary” child also progresses. Nevertheless, the very nature of training through drama performance suggests that there is a positive training effect.

From a methodological point of view, it is also necessary to mention the limitations of the case study.

First of all, as far as the interpretation of the data is concerned, we have, on the one hand, proceeded to a qualitative treatment of the data, which usually supports a quantitative treatment (BRIEF, LEAS-C). It seems that the productions obtained (answers written by the participant for the LEAS-C or by the teacher for the BRIEF) allowed this finer and more specific processing inherent to the case study. On the other hand, we decided to rely on the skill repositories to triangulate the previous data with those from the videos and interviews with double-blind analyses in order to avoid interpretation bias by proceeding with a meticulous description of the specificity of Charles’ behaviours in the situation. In this respect, Fortin’s Creative Skills Development Analysis Tool was very useful, as it lists relevant indicators of creativity for artistic performance.

However, despite our rigour, we believe that it is necessary in the future to complement our measurement of creativity with the use of a tool such as the EPoC (Evaluation of Children’s Creative Potential) test, which assesses divergent-exploratory and convergent-integrative thinking in the graphic and verbal domains (Besançon et al. 2011), as creative processes can vary not only between children but also within children depending on the domains considered (Barbot et al. 2017). In addition, the results show the importance of taking into account emotional prosody as a marker of the meaning given to speech. To this end, a prosodic reception test and the development of an adapted verbal production test to measure more precisely the development of the participants’ language skills are to be implemented for the next phase. We also believe that it is necessary to test the effect of the performative approach on a larger sample. Therefore, we have started the second pilot phase of the CELAVIE study, with the same objective, according to a test-re-test research design, this time with an experimental group practicing performance theatre (*n* = 23) and a control group (*n* = 24) of children of the same age group and from the same city, attending school in a neighbourhood, and practicing English through table games.

In conclusion, this first phase of the CELAVIE research was very useful in that it allowed us to verify the feasibility of the study. The case study with Charles allowed us to refine the methodology of data collection and analysis in order to answer our research questions in the most controlled way possible, despite our desire to conduct the study in an environmentally friendly way so as not to take the students out of their daily formal learning environment. Despite the few limitations identified at this stage of the study, the initial results are promising and encourage us to extend our research with the necessary adaptations.

## Figures and Tables

**Table 1:** Mixed data collection.

Pre-Test	during the Experiment	Post-Test	
- Executive functions (BRIEF, Gioia et al. 2013) - Cognitive-emotional skills (LEAS-C, Bajgar et al. 2005, semi-directive interviews with teachers) - Creativity (semi-directive interviews with teachers) - Oral production (audio-video recording with one camera from the language test in the classroom; semi-directive interviews with teachers). (See Section 3.4).	- Cognitive-emotional skills, creativity and embodied Language (Audio-video recordings * during the 4 workshops with the actor: 8 sessions)	- Cognitive-emotional skills, creativity and oral production (BRIEF, LEAS-C, testimonials of the teachers + of the actor, semi-directive interviews with the teachers and with the participant)	

* We used 2 Canon XF105 cameras (A and B). The first one (A), static, was installed in a corner of the room. This camera was capturing wide shots. The second one, mobile, was shooting close-up, focused on the target child. Both cameras were synchronized. The microphone of Charles was wireless. All the audio streams were synchronized on both cameras, using a clap. These audio-video data help us to understand the links between the behaviour (language embodiment) and the oral production of the participants.

**Table 2:** Charles’ BRIEF raw scores (teacher form): evolution of the raw scores of the behavioural regulation of Charles in pre- and post-test.

	Pre-Test	Post-Test
Inhibition	22	16
Flexibility	29	26
Emotional control	24	17
Behavioural regulation index	75	59

**Table 3:** BRIEF mean scores and standard deviations of the scales and indices for boys by age 7–10 for the BRIEF teacher form (French data) (Gioia et al. 2013, p. 53).

	Mean	Standard Deviation
Inhibition	14.05	4.47
Flexibility	12.46	3.00
Emotional control	11.17	3.78
Behavioural regulation index	37.68	9.75

**Table 4:** BRIEF mean scores and standard deviations of the scales and indices for boys by age 11–14 for the BRIEF teacher form (French data) (Gioia et al. 2013, p. 53).

	Mean	Standard deviation
Inhibition	12.53	3.86
Flexibility	12.08	2.87
Emotional control	10.43	2.99
Behavioural regulation index	35.08	8.39

**Table 5:** Qualitative assessment of Charles’ creativity according to the indicators of the creating skills in performing arts repository based on Fortin’s reference framework, before and after training.

**Creating Skills**	
1—Quality of presence to oneself, to the world, to experience (indicators: physical, emotional and intellectual availability; imagination; listening, observation; acceptance of the new, of the unknown).	
**Before training**	**After training**
**Low quality of presence** (a) Awareness of himself - Charles’s **emotional control** is well below the average for his age group (see Table 2, Table 3 and Table 4). - Charles shows a medium average of **intra-individual awareness** (see Figure 3) - **Charles frequently refuses to take part in activities**, when a situation is unusual and he feels upset: disengagement in the task (see Figure 4 and Figure 5**a**). The actor interprets this blockage quite positively: “he was equally quick to assess those moving around him and change his own actions when realising he was not ‘correct’. And equally quick at removing himself from an activity or situation that he didn’t like—this quality was strong from the start and stayed until the end and an important part of his personally creative process because it gave him the chance to express and understand what was bothering him, which in turn he could use in his performance. For example: the emotion in his voice” (actor testimony). - (b) Awareness of others and the environment - Charles shows a **low average of inter-individual awareness** (see Figure 3)	**Higher quality of presence** (a) Awareness of himself - Charles’s emotional control improves significantly (see Table 2, Table 3 and Table 4) - When he feels upset: Charles needs to talk to an adult for reassurance, but he resumes activities (re-engagement. See Figure 4 and Figure 5b). - “He can get angry quickly but calms down just as quickly when the adult listens to him and decodes the situation” (teacher testimony). - Observation of his behaviour on the videos shows that during the last session, when he was feeling very angry after an argument with a classmate, Charles still chose to participate in the activity but interpreted his character as angry. He now seems **able to use his emotion** (in this case anger in the last session) **as an aesthetic object**. (b) Awareness of others and the environment - Charles’ average of intra-individual awareness remains more or less stable but his **inter-individual awareness doubles**.
2—Quality of the exploration of the postural, mimicry and gestural expression (indicators: risk-taking by creating new worlds: ability of students to create and express themselves with the body; appropriation of instructions; commitment)	
**Before training**	**After training**
**Low quality of exploration** - “**Playing with** the volume of his voice, his posture or in role-playing activities and/or debates **is difficult for Charles**” (interview with the inclusion teacher). - The researchers observe that he **lets himself be guided** by his peers (indirect observations through the audio-video recordings from the language pre-test and drama sessions 1 and 2) - He **reproduces** patterns (repeats) (indirect observations through the audio-video recordings from the language pre-test) - He **imitates** his classmates rather than to propose something (indirect observations through the audio-video recordings) - “He says he loves American culture but gets angry quickly when he doesn’t understand what is being asked of him in English” (Teacher testimony). - “Charles is inactive if not solicited” (teacher testimony).	**Higher quality of exploration** - Analysis of the audio-video recordings of the final workshop sessions (7 and 8) showed that Charles was making suggestions at the end of the project, unlike at the beginning. **He makes scenic proposals**, both in terms of postural and gestural acting, and in terms of the emotional interpretation of the character being played. - “Charles let **his own idea** (ex. his version of a bench) inspire **a new idea** (ex. Driving a car?) showing **fluid creativity** and openness to letting it evolve” (actor testimony). - “He smiles; he is **engaged**, accompanied by another pupil, to prepare something to show to the others” (teacher testimony). - “[After the experiment], during the circle activities at the beginning of the lesson in class, **he can show initiative**—often when other pupils have already done so (e.g., instead of passing the flower to his neighbour with the sentence ‘I give you a flower’, **he mimes** throwing it on the ground to trample it—**with a big smile**) (teacher testimony). - The actor writes: “I noticed that by the end of the project, in moments of distraction, his impulsive actions were more often away from other classmates, **exploring gestures** alone, rather than exciting/distracting others. At the start of the project his creativity came from his interactions with others, but towards the end it came more from within, from **his own ideas**. He **became more and more expressive with his arms,** whereas start he was more constricted, perhaps reluctant to engage his whole body” (actor testimony).
3—Quality of the organisation of time, space and materials for creation (indicators: anticipation of the whole project; taking initiatives)	
**Before training**	**After training**
**Low quality of organisation** His material organisation’s score by the BRIEF test is low (83 points).	**Low quality of organisation** Despite a very slight improvement in the score (79 points: reversed scores), the material organisation’s score from Charles did not change significantly. We do not have sufficient data to position Charles’ behaviour on this indicator. At this stage, it seems that **Charles is not more able to anticipate future events, to plan ahead, to put in place appropriate** measures in advance to carry out an action or task, or to carry out tasks in a more systematic way.
4—Quality of collaboration with partners (indicators: ability of students to adapt their behaviour; contribution to teamwork; establishing a relationship climate; listening to the ideas of others)	
**Before training**	**After training**
**Low quality of collaboration** - “Charles is **inactive** if not solicited” (Teacher’s testimony) - His interpersonal awareness is **well below average** (see Figure 3) - His body engagement rates show a **disengagement by performing activities** in the first drama sessions (see Figure 4). - Charles says, he learned English **at home** with Google translate and he does not mention any interaction with his peers or learning situations in the classroom, even though he has been learning English at school for 3 years. (Charles’ semi-directive interview).	**Higher quality of collaboration** - Charles improves his **emotional awareness** scores, especially **towards others** (see Figure 3). - His **behavioural regulation (inhibition, flexibility and emotional control)** improves significantly (see Table 2, Table 3 and Table 4). - He interacts with peers (audio-video observations): “**he communicates** with some of the students in the class (male or female), he is **willing to work in a group**” (Teacher testimony). - His **engagement by performing activities** increases significantly (see Figure 4).
5—Quality of reflection on one’s work and the group’s work (indicators: analysing one’s own work and that of others; verbalising opinions; consideration of comments and criticism; renewal of one’s representations and perceptions)	
**Before training**	**After training**
**Low quality of reflection** - The audio-video recordings of sessions 1 and 2 show that Charles often **refuses to answer questions.** - He accepts the proposals from others but **seems to be passive** (audio-video recordings of sessions 1 and 2)	**Higher quality of reflection** - He accepts most of the time to **answer questions** (audio-video recordings of sessions 7 and 8) - **He proposes scenarios** to the group (audio-video recordings of sessions 7 and 8)
6—Quality of the formatting of the work (indicators: quality of the role play; interpretation)	
**Before training**	**After training**
**Low quality of formatting** - No gestures (pre-test of language) - No gestures and no facial expression, except when he gets angry. No scenic proposals	**Higher quality of formatting** - The auditory analysis of the prosodic curve shows a **slight improvement in the post-test compared to the pre-test**. The results of the language post-test also show a slight increase in the rate of speech and a more spontaneous reaction in the proposals. **He expresses more feelings** in his melodic curve than in the pre-test, where the prosody remained monotonous despite emotional content (“I am happy”). - **He makes more gestures and shows more facial expression** (observations from the teacher in the classroom, observations in the sessions 7 and 8)
7—Quality of the verbal expression (indicators: ability of speakers to produce new language appropriate to the context, originality of the verbal language, i.e., new words or new combination of words)	
**Before training**	**After training**
**Low quality of verbal expression** - Charles **does not speak spontaneously** in class. He **reproduces models** without autonomous proposals (teacher testimonial and video-observations) - **Prosody:** Typical of French intonation, Charles’ intonation curve is flat (auditory analysis of the language pre-test).	**Higher quality of verbal expression** - In classroom exercises after the training, **his English fluency**, as “the ability to produce several original responses quickly” (Monette and Bigras 2008, p. 325), **has improved** both behaviourally (gestures and facial expression) and verbally (teacher testimony). - Nevertheless, in the post-test situation, Charles still produces only one sentence and relies a lot on the proposals of his peers. - He uses **better prosody** in the drama-sessions too: “sometimes, he is just mumbling, and **sometimes with an excellent accent**” (actor testimony). - He shows more **autonomy** and **spontaneity** in verbal production regarding the academic expectations, after the performative theatre training. The teacher notices: “I think the drama practice was an opportunity for Charles to be required to communicate with others, to have to expose himself to the gaze of others. [It was for him the opportunity] to realise that he can do what others do and to realise that the gaze of others can be benevolent” (teacher testimony). - “Despite his hunger to understand why we did certain things and what it meant, **he never hesitated to speak (or sing!) in English**, sometimes just mumbling, and **sometimes with an excellent accent**” (actor testimony). - Charles produces **humour** using English: “When he was supposed to give a flower to a friend, he handed her the imaginary flower and, at the last moment, mimed crushing it under his foot and told her aggressively “I give you a flower!” He then begins to laugh, enjoying the surprised laughter of his friends” (teacher testimony). - “He **transforms** sometimes sentences to **create** new ones (e.g., can you play tennis?>Can you play Fifa22)” (teacher testimony) - “[he] has **memorised** simple English phrases and uses them appropriately”, “[he is] able to ask a question spontaneously (in English)” (teacher testimony).

**Nota bene:** The bold highlights in the textual quotations correspond to the coded elements of the corpus, which allowed us to sort them into the categories corresponding to the 6 observables of creativity. This coding was carried out in a double-blind setting.

**Table 6:** Positive trend in Charles’ scores: linking cognitive-emotional skills (BRIEF; LEAS-C) to creativity (Fortin et al.’s reference framework) and EFL in Charles’ case study.

	Emotions	Creativity
Cognition	- Improvement in emotional awareness (inter- rather than intra-awareness) (LEAS-C)	- Improvement in mental flexibility (subscale of the Behavioural Regulation Index, BRIEF). - Better quality of presence to oneself, to the world, to experience (better emotional awareness; better imagination); (Fortin’s et al. indicators of creative skills)
Behaviour	- Improvement in the commitment to the task (coding of body engagement with Elan). - Better inhibition and emotional control (subscales of the Behavioural Regulation Index)	- Better quality of presence to oneself, to the world, to experience (better acceptance of the unknown) (Fortin’s et al. indicators of creative skills). - Better quality of posturo-mimo-gestual exploration (Fortin’s et al. indicators of creative skills). - Better quality of collaboration with his peers (Fortin’s et al. indicators of creative skills).
Language (EFL)	- Better quality of verbal expression (improved tonal variation and accentuation; use of humour, transformation of linguistic models to create new sentences, improved fluency and rhythm of language) (Fortin’s et al. indicators of creative skills). - Better quality of posturo-mimo-gestual exploration (Fortin’s et al. indicators of creative skills).	

## Data Availability

Not applicable.
